# Combined Mucinous and Neuroendocrine Tumours of the Appendix Managed with Surgical Cytoreduction and Oxaliplatin-based Hyperthermic Intraperitoneal Chemotherapy

**DOI:** 10.7759/cureus.3894

**Published:** 2019-01-16

**Authors:** Roy Hajjar, Pierre Dubé, Andrew Mitchell, Lucas Sidéris

**Affiliations:** 1 Surgery, Université de Montréal, Montréal, CAN; 2 Surgery, Hôpital Maisonneuve-Rosemont, Montréal, CAN; 3 Pathology, Hôpital Maisonneuve-Rosemont, Montréal, CAN

**Keywords:** combined tumors, mucinous neoplasm, neuroendocrine tumor, perforated appendicitis

## Abstract

Appendiceal neoplasms account for 1% of appendectomy specimens. Common subtypes include mucinous cystadenoma, adenocarcinoma, and neuroendocrine tumors (NETs). The simultaneous presence of appendicular mucinous and NETs is a rare event. Depending on the tumors’ morphological distribution in the affected organ, they are qualified as either “collision” or “combined” tumours. We herein present the case of a 50-year-old male who presented with acute appendicitis and who was subsequently found to have pseudomyxoma peritonei (PMP) due to a perforated combined mucinous and neuroendocrine tumours. The patient was treated by right hemicolectomy and cytoreductive surgery (CRS) with oxaliplatin-based hyperthermic intraperitoneal chemotherapy (HIPEC). He was cancer free 20 months later. Due to the limited clinical experience with this presentation, no formal recommendations exist as to its management other than those applicable to each cancer alone. The efficacity of treatment on the long-term prognosis on these combined tumors is yet to be elucidated.

## Introduction

Acute appendicitis affects mainly young individuals and may lead to perforation and abscess [1]. Appendiceal neoplasms are found in 1% of appendectomy specimens [2]. Common subtypes include low-grade mucinous lesions, adenocarcinoma, and neuroendocrine tumors (NETs) [2-3].

The simultaneous presence of neuroendocrine and mucinous neoplasms in the appendix is exceptional with only a few previously reported cases [4]. To the best of our knowledge, the clinical presentation of these synchronous tumours as perforated appendicitis with an abscess is a rare event, and there is little data regarding their management and long-term prognosis. We herein present the case of a 50-year-old male who presented with acute appendicitis and who was subsequently found to have pseudomyxoma peritonei (PMP) due to a perforated combined mucinous and NET.

## Case presentation

A 50-year-old male presented to the emergency room with abdominal pain. An abdominopelvic computed tomography (CT) scan showed a perforated appendicitis with a contained abscess. Laparoscopic exploration revealed a neoplastic appendiceal lesion with peri-appendicular and pelvic mucin as seen in PMP. A laparoscopic appendectomy was performed. 

**Figure 1:**
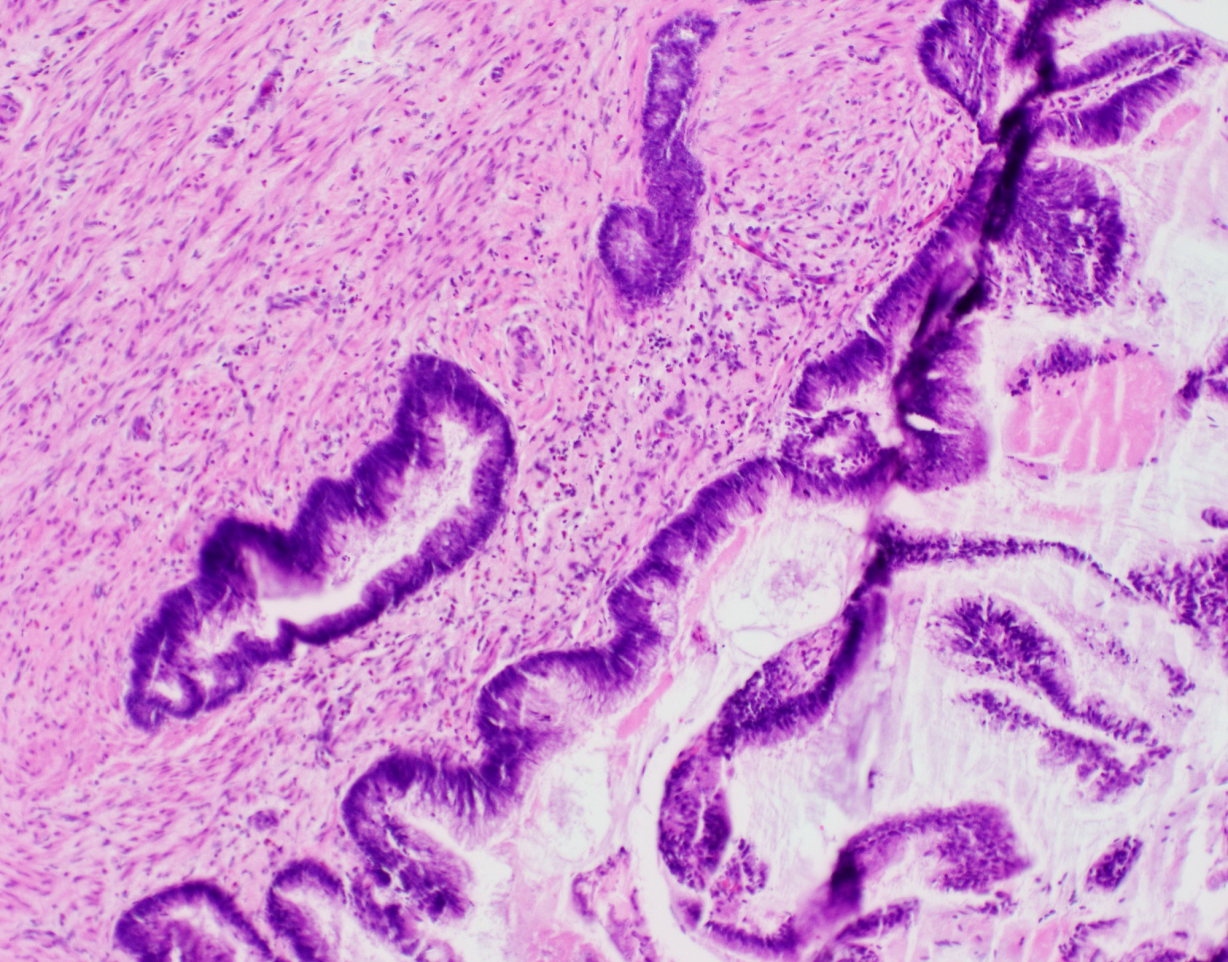
Medium-power magnification of low-grade appendiceal mucinous neoplasm (LAMN) There is minimal cytological atypia of the epithelial cells.

**Figure 2:**
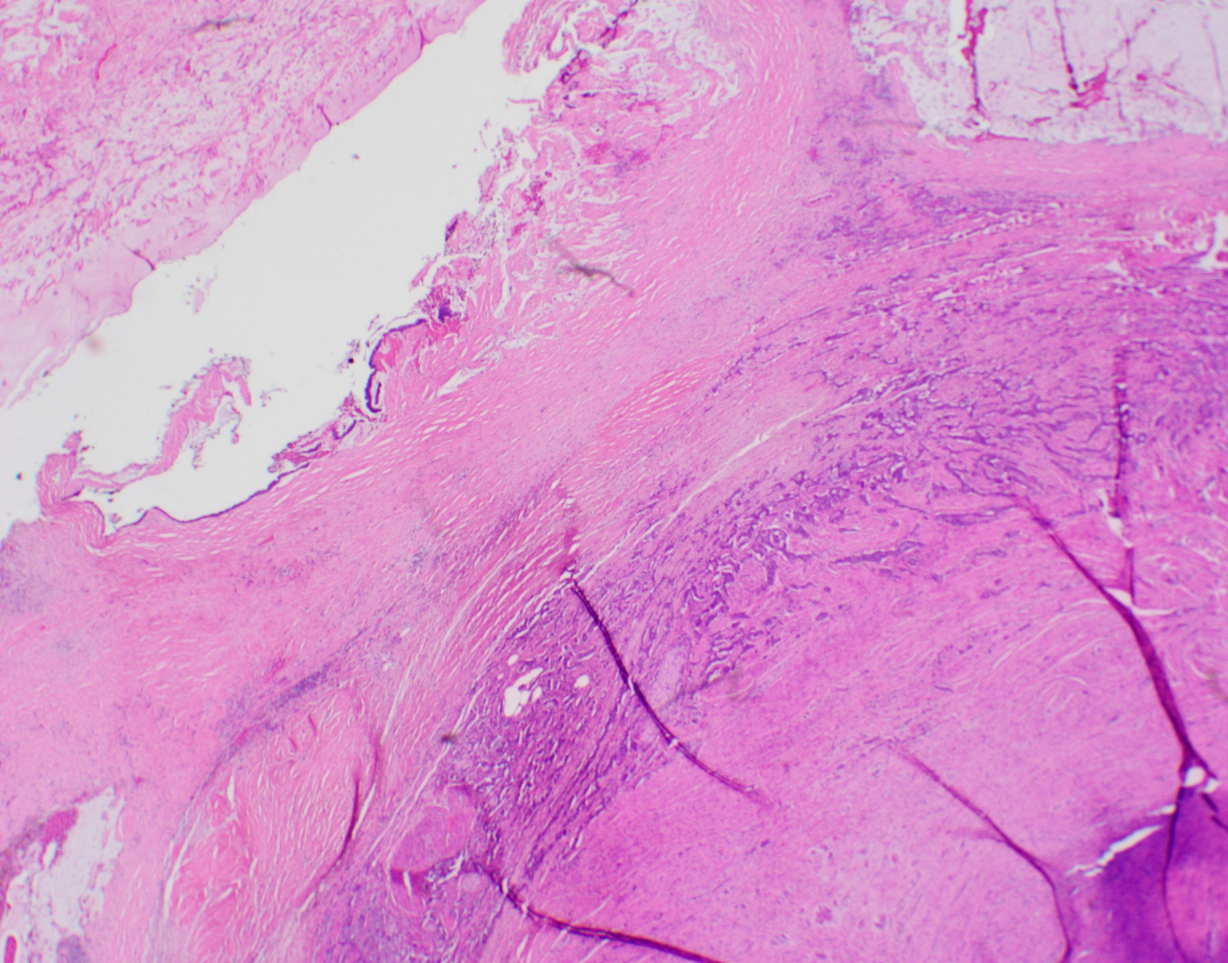
Low-power magnification of the appendix wall showing combined low-grade appendiceal mucinous neoplasm (LAMN) (left) and well-differentiated neuroendocrine tumor grade 1 (right). The latter is composed of cords and tubules of neuroendocrine cells

Pathological examination revealed the coexistence of mucinous and neuroendocrine appendicular tumours (Figures 1-2). The former corresponded to a low-grade mucinous adenocarcinoma which had developed from a low-grade appendiceal mucinous neoplasm (LAMN). The tumor was 5.5 cm long and occupied the entire appendix. A perforation site was identified as well as neoplastic cells in the lumen of the resection margin. The second tumour was a well-differentiated NET measuring 1.6 cm, with infiltration of the muscularis propria and minimal infiltration of the mesoappendix. The proliferation index as evaluated by the immunohistochemical marker MIB-1 was approximately 3%, corresponding to a histologic grade of G2/3. Perineural invasion, but no vascular invasion, was visualized. The resection margin was negative for NET. No lymph nodes were identified in the appendectomy specimen.

A complete workup, including thoracic and abdominopelvic CT scans, total colonoscopy, and evaluation of serum markers (carcinoembryonic antigen and chromogranin A) was normal. As the prognosis was considered more likely linked to the PMP rather than the NET component, right hemicolectomy and cytoreductive surgery (CRS) with hyperthermic intraperitoneal chemotherapy (HIPEC) were performed five months after the appendectomy.

At laparotomy, there were mucinous lesions on the peritoneal surfaces of the right hemidiaphragm, right abdominal wall, greater omentum, right colon, and pelvis. The peritoneal carcinomatosis index (PCI) was 22. Right hemicolectomy was performed as well as total omentectomy, cholecystectomy, and peritoneal stripping of the right hemidiaphragm, abdominal wall and pelvis were performed. A completeness of cytoreduction score of 0 – with no residual peritoneal seeding – was achieved. HIPEC with oxaliplatin (460 mg/m^2^) for 30 minutes at 42°C was then administered.

Histopathologic examination revealed the presence of acellular mucin in the ligamentum teres hepatis, the peritoneal surface of the right colon, greater omentum, and Douglas' pouch. A suspicious right colic artery lymph node was analyzed intraoperatively and was found to be covered by acellular mucin. The right diaphragmatic peritoneal surface displayed mucinous epithelial cells with high-grade dysplasia. The right hemicolectomy resection margins and 17 lymph nodes were cancer-free. The patient recovered uneventfully after the surgery and remains cancer-free after 20 months of follow-up.

## Discussion

Appendicular NETs account for <1% of gastrointestinal neoplasms [3-4]. They are usually located in the distal appendix. Considering their ability to secrete vasoactive peptides, they can cause flushing and diarrhea, a presentation known as the “carcinoid syndrome”. NETs <2 cm rarely metastasize and are associated, if no nodal or distant disease is present, with a five-year survival of >90% [5]. The suggested mainstay of treatment is a right hemicolectomy for tumors >2 cm, and for those <2 cm having a vascular or mesoappendiceal invasion, positive margins, or mixed histology [5]. Appendectomy seems to be otherwise sufficient.

Mucinous tumours of the appendix include LAMNs, high-grade appendiceal mucinous neoplasms, serrated polyps, adenomas, and adenocarcinomas [6]. Mucin secretion by these tumors can lead to appendiceal rupture and dissemination of tumoral cells in the peritoneal cavity. The resulting implants could be cytologically benign, originating from mucinous adenomas, and form what is best known as “disseminated peritoneal adenomucinosis” (DPAM), or contain malignant cells and induce mucinous carcinomatosis [7]. The overall five-year survival rate with these entities after CRS and HIPEC is 81% and 59% respectively [8].

The coexistence of mucinous and neuroendocrine appendiceal tumors is an exceptional event with only a few well-described cases having been published [4]. These may occur as “collision” tumors or as “combined” tumors. Collision tumors are adjacent to each other but with well-demarcated margins and a featureless stroma in-between [9]. Combined tumors have intermixed cell populations [9].

It is worth noting that the management of NET-derived peritoneal carcinomatosis (PC) is the subject of ongoing debate. The literature on the optimal management of this tumour's rare presentation remains scarce. Due to the significant morbidity associated with HIPEC, surgical cytoreduction alone has been suggested as a valid option to treat NET-derived PC. However, it is not established if the combination of CRS and HIPEC is the best therapeutic approach in such a situation [10]. Nonetheless, if our patient had also displayed peritoneal neuroendocrine metastases, we believe HIPEC would have been indicated knowing the significant therapeutic advantage it provides in the management of the PMP component. Although the efficacy of such an extensive procedure depends substantially on several non-modifiable factors, such as the grade of the mucinous neoplasm and the associated PCI, aggressive CRS and HIPEC remains the mainstay of a curative surgical approach with regards to combined mucinous and neuroendocrine appendiceal tumours with peritoneal dissemination [11-13].

Due to the rareness of these combined tumours and the lack of experience of HIPEC use for mucinous neoplasms in the presence of a NET, postoperative follow-up guidelines are at present difficult to establish. Nonetheless, monitoring via imaging studies and tumour markers personalized to the patient’s risks and tumoral histological type seems reasonable [4].

## Conclusions

In conclusion, combined appendicular mucinous and neuroendocrine neoplasms with PMP are extremely rare. We believe that management using aggressive CRS and HIPEC will provide the greatest possible impact on prognosis. Further experience in the treatment and follow-up of these tumours is necessary to validate this approach.
